# Effectiveness of Interventions to Improve Vaccination Coverage in India: A Systematic Review and Meta-Synthesis of Qualitative Studies

**DOI:** 10.7759/cureus.97111

**Published:** 2025-11-17

**Authors:** Aviraj KS, Neethu George, Neeraj V Mohandas, Rock B Dharmaraj

**Affiliations:** 1 Community Medicine, Employees' State Insurance Corporation (ESIC) Medical College & Hospital, Noida, IND; 2 Community Medicine, Dhanalakshmi Srinivasan Medical College Hospital, Siruvachur, IND

**Keywords:** community engagement, interventions, meta-synthesis, qualitative studies, systematic review, vaccination, vaccine hesitancy

## Abstract

This is a systematic review and meta-synthesis of qualitative studies to identify the facilitators and barriers of interventions aimed at improving vaccination coverage in India, along with exploring the beliefs and practices regarding vaccination. The focus was on interventions targeting beneficiaries, healthcare providers, and the health system via recipient-focused methods, provider-oriented training, and health system reforms to increase vaccination coverage. Search terms were adapted from database scoping and title, abstract, and index term analysis, and a thorough literature search was conducted in PubMed, Scopus, and Crossref databases for studies published in English on the topic of research between January 2013 and December 2024. Among the 12 articles selected for the study, four were mixed-method studies, and five were conducted among stakeholders in the community providing the vaccination. The quantitative synthesis revealed several key themes: beneficiary-related (improved vaccine acceptance), community-related (mobilization, awareness, ownership, and leadership), and healthcare provider-related (better stock management, endorsement, and engagement). Additional themes included local advocacy and the integration of family planning with infant vaccination. Barriers were categorized as system-related (e.g., accessibility issues, coercive strategies), information-related (misinformation, lack of awareness), and logistic-related (limited resources, indirect costs). Effective interventions were found to leverage community engagement, improve healthcare provider practices, and enhance vaccine acceptance among beneficiaries. However, significant barriers persist, including systemic challenges, information gaps, and logistical constraints. Social norms, health attitudes, and trust in healthcare systems have emerged as critical factors affecting vaccination uptake. These findings underscore the need for multifaceted, culturally sensitive approaches to improve vaccine coverage in India. Future interventions should address identified barriers while building upon successful strategies to ensure more comprehensive and sustainable vaccination programs.

## Introduction and background

The Declaration of Alma-Ata, adopted by the World Health Organization (WHO) in 1978, established the foundational principles of primary healthcare, calling for coordinated efforts by governments and the global community to ensure health for all citizens. Essential to this approach is the prioritization of vaccination against infectious diseases [1,2]. Since then, vaccination has evolved into a crucial public health measure, alleviating the burden of numerous vaccine-preventable infectious diseases and reducing healthcare expenses [3].

The history of vaccination in India reflects a journey marked by significant milestones, from the introduction of smallpox vaccination during the British colonial era to the establishment of comprehensive immunization programs addressing a range of preventable diseases [4,5]. In 1978, India adopted primary healthcare concepts, such as immunization, in accordance with the Alma-Ata Declaration. The Universal Immunization Program (UIP) was initiated in 1985 to offer free immunization services across the country with the goal of protecting children from vaccine-preventable diseases and reducing morbidity and death rates. India has progressively broadened its national vaccination schedule to incorporate vaccines for diseases such as hepatitis B, *Haemophilus influenzae* type b (Hib), rotavirus, pneumococcal disease, Japanese encephalitis, and, most recently, COVID-19. Through focused and persistent actions, significant accomplishments have been made, such as eliminating diseases such as polio and making advancements in managing diseases such as measles and tetanus [6,7].

Data from the National Family Health Survey (NFHS) 2019-21 (NFHS 5) revealed that child vaccination coverage in India has reached 76.4%. While this represents a notable improvement of 14.4% compared with the NFHS-4 (2015-16), It is crucial to note that the goal of achieving 90% immunization coverage (by 2030 according to WHO Immunization Agenda) among children remains unmet [8,9]. Among older people, vaccination coverage is less than 2%. The reported vaccination rates are 1.5% for influenza, 0.6% for pneumococcal illness, 1.9% for typhoid, and 1.9% for hepatitis B. With regard to COVID-19, India has achieved 100% first-dose coverage and 80% full immunization coverage by administering a total of 1.7 billion vaccine doses to over 940 million persons [10,11].

The suboptimal vaccination rates, despite gradual improvements, may be explained by a range of interconnected variables, both internal and external. Limited accessibility due to challenges such as distance, transportation issues, financial barriers, and inadequate healthcare infrastructure plays an important role. Factors such as difficult waiting conditions or discouraging settings can lead to vaccine hesitation. Social effects such as negative feedback from friends, cultural and religious systems, and socioeconomic factors such as lack of education and health literacy also contribute to it. Vaccine hesitation also arises from safety concerns, inadequate awareness of the importance of immunization, and beliefs about decreased disease risk [12-17].

To improve vaccination coverage, several initiatives have been developed that focus on various stakeholders, such as beneficiaries, healthcare professionals, and the health system. Recipient-focused interventions utilize strategies such as communication, reminders, decision-making assistance, community participation, and incentives to increase the demand for vaccinations. Provider-oriented treatments involve training, feedback mechanisms, supervision support, and incentives to improve providers' knowledge, skills, and service quality. Health system interventions focus on enhancing service accessibility and supply through infrastructure development, logistic support, and service delivery methods [18-22].

Qualitative studies provide in-depth insights into the complex sociocultural, economic, and behavioural factors that influence vaccination decision-making in conjunction with quantitative research. Researchers use them to gather a wide range of perspectives and experiences from stakeholders, such as vaccine users, healthcare professionals, politicians, and community leaders. Qualitative research delves into the complex dynamics of these elements, uncovering concealed or unforeseen obstacles to immunization. It also helps in comprehending the difficulties and achievements of intervention implementation, which enhances delivery and sustainability. Qualitative research enhances the comprehension of the contextual complexities and operational processes of interventions, complementing quantitative data, and can address health inequities and advance social justice in immunization programs [23-32].

This review aims to identify the facilitators of and barriers to interventions aimed at improving vaccination coverage in India. The review also explores the beliefs and practices regarding vaccination among different populations in India.

## Review

Methodology

A meta-synthesis technique was used for this qualitative review [33]. The protocol was registered in the International Prospective Register of Systematic Reviews (PROSPERO) (registration no: CRD42024539989). The review was designed and conducted in accordance with the Preferred Reporting Items for Systematic Review and Meta-Analysis (PRISMA) 2020 checklist (see Appendix A) [34]. The research questions were: a. What are the facilitators of interventions to improve vaccination coverage in India? b. What are the barriers to vaccination coverage in India? c. What are the beliefs and practices regarding vaccination in India?

Eligibility Criteria

The review included qualitative and mixed methods studies on the following areas of interest: factors affecting vaccination coverage across the different regions in India, including the facilitators of interventions for vaccination coverage in the community, barriers to vaccination coverage and beliefs, and perceptions of stakeholders regarding various childhood and adult vaccinations across India. Only the qualitative component of mixed methods studies was considered. Studies published between January 2013 and December 2024, with the full texts available in English, were included. Only studies that utilized qualitative methods, including interviews, focus group discussions, and direct observations, were considered. All quantitative studies, including case reports, case series, reviews, pilot studies, conference abstracts, and expert opinions, were excluded.

Search Strategy

The search terms were identified and adapted from an initial search of databases and analysis of text from titles, abstracts, and index terms. This was followed by a systematic literature search in three databases (PubMed, Scopus, Crossref). Harzing’s Publish or Perish software [35] was used to search the abovementioned databases. The search was repeated via Google and Google Scholar to include gray literature.

The search terms included medical subject headings (MeSH), free words, and selected keywords. The search was tailored to the formats, operators, and conventions of each database via a variety of search terms related to the phenomenon of interest, context, setting, and qualitative methods (see Appendix B). The references of the included studies were searched manually for other relevant articles.

Screening of Studies

Following the search, all identified articles were collated and uploaded into the Rayyan software [36]. The duplicates were removed first, and as per the proposed eligibility criteria, title and abstract screening were independently conducted by two reviewers (NVM, AKS). The secondary screening included independent double screening of 10% of the abstracts and titles under the “included” section and repeated screening of all the articles under the “undecided” and “excluded” sections in Rayyan. The requirement for the inclusion of articles was agreement between both reviewers. Based on this screening, a list of articles eligible for full-text review was prepared. The reasons for the exclusion of full-text articles were noted and reported in the systematic review. Conflicts among the reviewers were resolved by mutual agreement or by the third reviewer (NG).

Methodological Integrity and Validity Checks

Interrater reliability measures (Kappa) between NVM and AKS were performed while screening and selecting studies to ensure that the inclusion criteria were clearly stated and that the articles were assessed equally. A consensus process was chosen to foster multiple interpretations, and any disputes between the authors were resolved by a third reviewer (NG). A critical auditor (RB) was selected to review and provide detailed feedback on each stage of the analysis and writing process.

Risk of Bias Assessment and Appraisal of Quality

Qualitative checklists were used to improve the transparency of all aspects of qualitative research by providing clear standards for reporting qualitative research. The Critical Appraisal Skills Program (CASP) checklist [37] was used for risk bias assessment. All 12 included studies achieved excellent total scores (see Appendix C).

The Standards for Reporting Qualitative Research (SRQR) checklist [38], a validated tool, was used to determine the methodological quality of the included articles in the review. The checklist consists of 21 items that are considered essential for complete, transparent reporting of qualitative research (see Appendix D). Two reviewers (AKS and NVM) independently read, reread, and evaluated the articles.

Data Extraction

Study information, such as author, year of publication, study area, type of sampling, sample size, age group of the study population, beliefs and practices regarding vaccination, facilitators of intervention, and barriers to vaccination, was extracted via a phased approach and represented in Microsoft Excel (Microsoft Corporation, Redmond, Washington, United States). Thematic synthesis was chosen as the qualitative evidence synthesis method [39]. A theme is a construct “that captures something significant about the data in relation to the research question and represents some level of patterned response or meaning within the dataset” [40].

Using an inductive approach, the authors extracted the data by reading and rereading the articles to familiarize themselves with the content, followed by sorting the data according to characteristic themes and subthemes of the main outcome variables and additional outcome variables. Codes were developed based on patterns and similarities in the themes and subthemes. Themes were considered only if there were two or more codes underlying the theme. The preliminary coding scheme was developed in NVivo software version 14 (Lumivero, LLC, Denver, Colorado, United States) [41], where line-by-line coding was performed inductively and iteratively by the authors (AKS and NVM), allowing an initial synthesis of information. The codes were collated, analysed, grouped, and categorized into narrow sets of codes, which were then used for interpretation.

Data Synthesis and Analysis

One author (AKS), with experience in qualitative methods, conducted the qualitative meta-synthesis via R software v4.1.2 (2021; R Foundation for Statistical Computing, Vienna, Austria, https://www.R-project.org/), in which the most common themes, subthemes, verbatims and codes regarding the facilitators of intervention, barriers to vaccination coverage and perceived beliefs and practices regarding vaccination were collated, analysed and categorized on the basis of statements and ideas across data. Sentiment analysis was performed to represent the beliefs and practices regarding vaccination from the beneficiaries’ and stakeholders’ perspectives, and was represented via a tree map. Codes representing unique sentiments towards vaccination were quantified, and the codes that appeared more than once were included. Anonymized sentiments from the participants were translated into a numeric score to quantify their positions regarding vaccination. Randomized sentiment scores were generated to represent a hypothetical range of attitudes from negative to positive for illustrative purposes, and scores were assigned to each code to aid in tree map visualization.

The tree map shows the frequency of each code and its corresponding sentiment score via a colour gradient. The colour coding indicated a wide range of sentiments, from deep concerns to strong endorsements of vaccination practices. A Sankey diagram was constructed to visualize the flow of themes from broader categories to more specific subthemes and codes. It illustrates the connections between different levels of analysis in a hierarchical structure. Subgroup analysis based on time and place has been performed to identify temporal and geographical patterns in intervention effectiveness and to understand cultural/socioeconomic and cultural impacts. To improve the overall relevance, two reviewers (NG and AKS) reread the primary articles, searched for illustrative quotes, and wrote the findings. In the end, the tentative model of the findings with the quotes was sent to another reviewer (RB), who served as a critical editor to assess the interpretations.

Results

**Figure 1:**
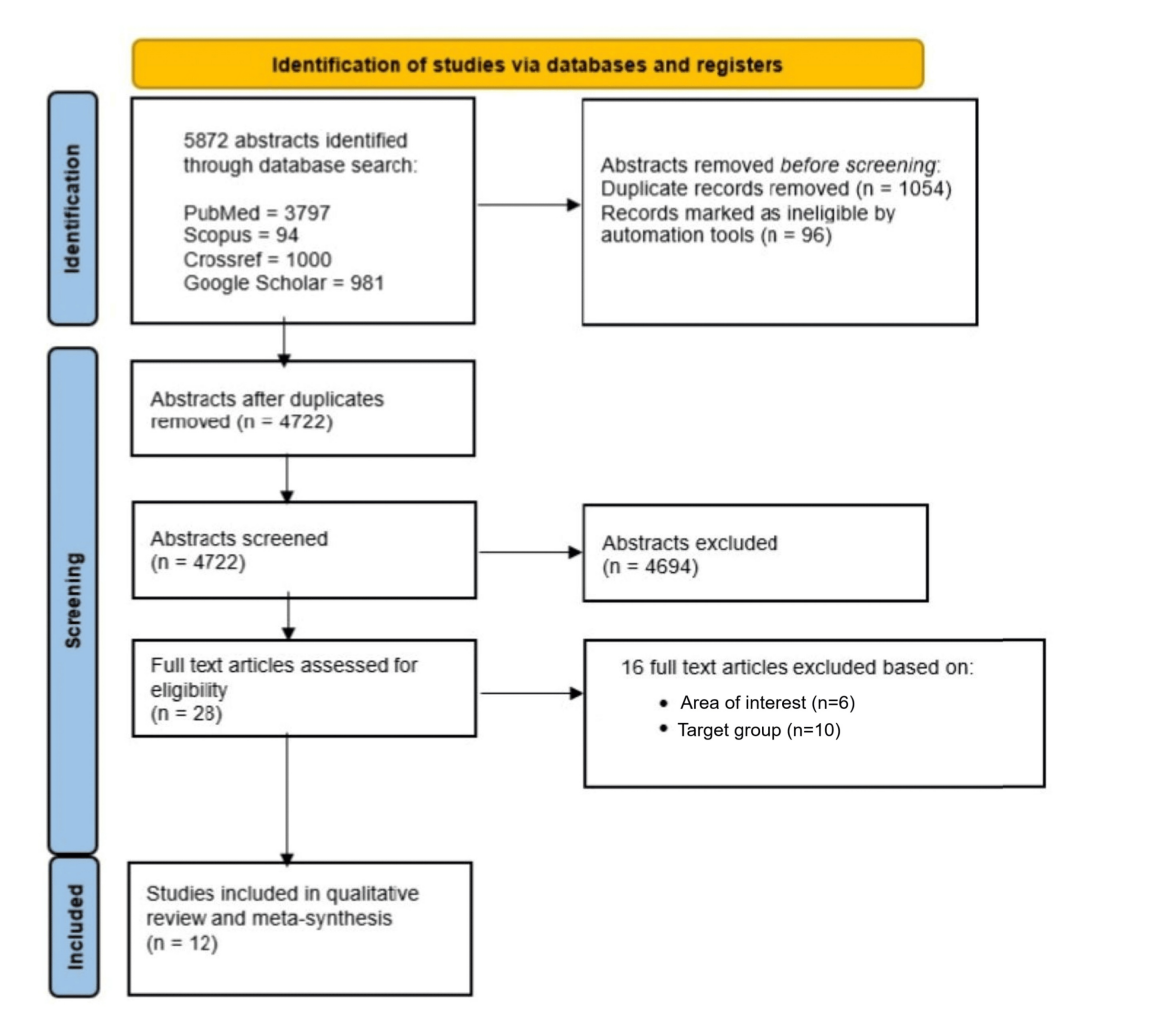
PRISMA flow diagram PRISMA: Preferred Reporting Items for Systematic Reviews and Meta-Analyses

In this study, 12 qualitative articles were selected to address the research question. A total of 5872 abstracts were identified through a database search, and among these, 28 full-text articles were assessed after eligibility. Finally, a total of 12 articles were selected. The PRISMA flow diagram is given in Figure 1.

A summary of the demographic characteristics of the studies and methodology-related factors is given in Tables 1, 2. Twelve studies were included in this meta-analysis, four of which [42-45] were mixed methods studies and the others [46-53] were qualitative studies. The analysis was presented in broad headings, themes/subthemes for facilitators and barriers, interconnected influences of facilitators and intervention sentiment analysis in vaccination: beneficiaries and stakeholders' perspective, analysis of beliefs, myths, and practices affecting vaccination.

**Table 1:** Demographic characteristics of included studies

S. No.	Year	Author	Study area	Study setting	Study population	Age in years	Male: Female	Education
1	2023	Averbach et al. [49]	Rural Maharashtra	Rural infant vaccination camps	Postpartum married women, husbands, and mothers-in-law of postpartum women, frontline healthcare workers and Accredited Social Health Activists, and community leaders	Postpartum women were 20–31 years old. Participating husbands were 27–42 years old.	1:3	-
2	2016	Sengupta et al. [45]	Ludhiana, Punjab, India	Slum settlement	Community members, auxiliary health care workers, community leaders, medical practitioners (alternative/allopathy)	-	1:2	-
3	2020	Giduthuri et al. [48]	Urban Pune, India	Middle-class and slum communities	Pregnant women and their spouses in slum and middle-class areas; private clinicians providing antenatal care (ANC)	-		Community sample Women-90% ≥10 years of Education Spouses-3.3%, ≥10 years of Education 100% employed. Clinician sample: 12-obstetrics and gynecology, 4- general practitioners
4	2024	Godbole et al. [53]	Six states in India: Uttar Pradesh Tamil Nadu, Maharashtra, West Bengal, Assam, and Chhattisgarh	-	Adult population across six different states in India	18 years to 72 years	1:1	Education- illiterate to those with primary, secondary, high school, and even graduation levels of education. Occupation- students, homemakers, unemployed, retired, unskilled workers such as manual laborers and farmers, and skilled workers such as carpenters and businessmen
5	2013	Paul et al. [51]	Andhra Pradesh, India	Population-based cervical cancer screening Study group, the Community Access to Cervical Health (CATCH) Study	Parents with daughters under 18 years of age	19–62 years old (average = 30)	1:5	Education -none to postgraduate. degrees, Occupation-67% employed
6	2013	Lahariya et al. [42]	Punjab, Madhya Pradesh, West Bengal, Karnataka, and Tamil Nadu	5 states (2 districts) in India	State Immunization Officers, District Immunization Officers, cold chain officers and handlers, pediatricians, both in public and private sector, and of vaccinators/Auxiliary Nurse Midwives,	-	-	-
7	2019	Krishnamoorthy et al. [44]	Puducherry, India	Rural health centre of tertiary institute	Parents of children	Middle aged parents	1:1	-
8	2023	Umesh Kawalkar et al [50]	Rural area of Akola district, Maharashtra	Rural and tribal area	Medical officers	45.04 (6.9) years	3:2	16 (53.3%)- MBBS degree
9	2022	Dhaliwal et al. [46]	Mewat District, Haryana	-	Caregivers-11 mothers, 1 father and 1 grandmother of children	-	-	-
10	2023	Dhaliwal et al. [47]	Mewat District, Haryana	-	Community Health Workers (CHWs), Community Accountability Board (CAB) members, parent and child caregivers	-	-	-
11	2013	Varghese et al. [52]	Kerala and Tamil Nadu, India	-	Stakeholders including healthcare providers, beneficiaries	-	-	-
12	2021	Das et al. [43]	Delhi, India	-	Visitors at a tertiary care hospital	-	-	-

**Table 2:** Methodology related details in studies

S. no.	Study	Type of study	Delivery mode of intervention	Type of intervention	Type of health care setting	Sample size	Tool for data collection	Type of sampling	Type of analysis (Thematic/Content)
1	Averbach et al. [49]	Qualitative study- key informant interviews	Community-based rural infant vaccination camps	linked family planning and infant vaccination care	Rural	60	Semi-structured interviews	Snowball sampling and purposive sampling	Content analysis
2	Sengupta et al. [45]	Mixed-method study	Community-based immunisation centres	Community-based intervention	Government-funded outreach clinics in non-notified slum communities	27	Interviewer-administered questionnaire	Stratified random sampling	Thematic analysis
3	Giduthuri et al. [48]	Ethnographic qualitative survey	Antenatal clinics	Educational and awareness-raising in the community cinics	Private clinics	106	Semi-structured interviews	Purposive sampling	Thematic analysis
4	Godbole et al. [53]	Exploratory qualitative study	Vaccination centres	Vaccination campaign	Primary Healthcare Centres	169	In-Depth Interview/Focus Group Discussions/Key Informant Interviews	Multistage purposive sampling	Thematic analysis
5	Paul et al. [51]	Qualitative study	Vaccination programmes in the state	socio-cultural environment and current vaccine infrastructure	Peri rural areas	36	Semi-structured interview	Purposive selection	thematic/content
6	Lahariya et al. [42]	Mixed-method study	Vaccination programme	Preventive healthcare - vaccination	Institutional deliveries, Primary Health Centres, session sites	143	-	Purposive sampling	Thematic
7	Krishnamoorthy et al. [44]	Explanatory mixed-method study	Vaccination centres	MR Vaccination campaign	Community-based	6	semi-structured questionnaire	Systematic random sampling	Thematic
8	Kawalkar et al. [50]	Qualitative study	Vaccination sessions	COVID-19 vaccination outreach program	Primary Health Centres in rural and tribal areas	30	Semi-structured interviews	Purposive sampling	Thematic
9	Dhaliwal et al. [46]	Qualitative study	Rural vaccination centres	social and economic benefits of Immunization	Rural healthcare setting, specifically Anganwadi Centres	13	Semi-structured interviews	Purposive sampling	Thematic
10	Dhaliwal et al. [47]	Qualitative study	Community-based participatory research	Community Health Worker-Led Intervention for Vaccine Information and Confidence (CIVIC) Project	Community-based	69	In-depth interviews	Convenience sampling	Thematic
11	Varghese et al. [52]	Descriptive and analytical qualitative method	Immunisation sessions	Immunisation services	-	-	Observations, focus group discussions (FGDs), and In-depth interviews	Three-step sampling	Thematic/content
12	Das et al. [43]	Mixed method	Educational and interactive sessions	Health education	-	668	semi-structured Questionnaire	Observational sampling	Thematic/content

Themes for Facilitators of Interventions

The quantitative synthesis identified 'improvement of vaccine acceptance' as the most frequently occurring theme, with a significant margin compared to other themes. The exact counts revealed a hierarchy of facilitators, with 'Community mobilization and awareness' and 'Community acceptance and compliance' following the second and third most prevalent themes, respectively. The themes with the verbatim are represented in Table 3. The 'improvement of vaccine acceptance' was observed in a broad range of contexts within the studies, often associated with successful communication strategies and public health messaging [43]. 'Community mobilization and awareness' was noted for its role in engaging local stakeholders and leveraging social dynamics to increase vaccine uptake [53]. 'Community acceptance and compliance' underscored the impact of cultural norms and the acceptance of vaccines within community networks, suggesting a correlation between communal practices and vaccination rates [52]. In a study by Sengupta et al., healthcare personnel felt that community people took ownership of the intervention, such as outreach immunization clinics/guardians, and were effective in increasing accessibility to and uptake of vaccinations in the community [45]. Averbach et al. noted the convenience of timing and location for families, along with programmatic support from governmental and community leaders [49].

**Table 3:** Themes for facilitators of interventions

Themes	Verbatims
Improvement of vaccine acceptance	"Awareness sessions helped us understand the importance of masks and social distancing" - by beneficiary appreciated health education efforts. "Through direct interaction, we could address misconceptions and fears about vaccines effectively" - by healthcare worker, discussing the impact of face-to-face education [43].
Community mobilization and awareness	A District Health Officer, high performing district - "ASHAs and ANMs played an important role in creating awareness among public by their household visits." Male participant from a high performing district who has taken the precaution dose, - “When we visited the temple, they used to announce to get ourselves vaccinated. This motivated us to get vaccinated" [53].
Community Acceptance and Compliance	A public health worker - "Trusted community health workers play a crucial role in educating parents and dispelling myths, thus improving vaccination rates." A Healthcare Provider - "Regular community meetings led by health professionals help to maintain transparency and build trust, acting as a facilitator for the immunization program" [52].
Improved stock management/Clear and timely central level instructions and oversight	A health official involved in private sector - "Involvement of private practitioners could help increase coverage as they participate in the vaccination process." A participant in a Stakeholder discussion on logistical support - "Regular and timely vaccine supply from the national level may help in smooth implementation of new vaccine introductions" [42].
Health provider endorsement	Father, 38 years old - "She [Auxiliary Nurse Midwife] says if we give the vaccine from childhood onwards then we can prevent certain diseases, so that’s why we’re prepared to give vaccines to children." A health Provider - "Health care personnel's recommendations and government endorsement play an integral role in vaccine behaviours" [51].
Strengthened community engagement.	Religious leader - "The involvement of local religious leaders who endorsed the vaccine was crucial. They helped to break down barriers and misinformation." A community Health worker - "Workshops provided by the project were effective in educating and empowering us to advocate for vaccination more confidently" [47].
Local advocacy and support	A Caregiver trusting local health workers - "Whenever they come for vaccination, we ask Sapna [Anganwadi worker] about it. We only go for it after asking her." Another participant - "My father-in-law is a doctor, so he vaccinated my oldest child. He shared that it is good to get it done" [46].
Clinician recommendation	A clinician in slum site - "Influenza vaccine is beneficial if pregnant women receive it ... It protects the mother as well as newborn". A person from middle class setting - "we should implement it; this is the only policy to protect pregnant women from severe types of influenza in their immune-compromised state" [48].
Role of healthcare providers and community engagement	An Auxiliary nurse Midwife - "Informing about the campaign in advance will give us more time in creating awareness about the importance of vaccine through routine home visits, Village Health Nutrition Days and special health education sessions." Another senor nursing officer - "IEC activities should be done more frequently and should address the need, safety and eligibility for the vaccine. This will reduce the confusion and hesitancy among the parents and will increase the coverage" [44].
Community leadership and engagement	A health care worker - "There should be proactive involvement of other than health department like for registration, teachers from primary school should be involved. Such involvement should not be on paper but actually on the field." Another health care worker - "We took help from the police department when people unnecessarily argued for the nonavailability of vaccine and created a panic situation that was difficult to handle by us" [50].

Subthemes for Facilitators of Intervention

Further analysis revealed subthemes that were crucial components of the broader themes. For example, within the realm of 'improving vaccine acceptance', subthemes included targeted education campaigns and the debunking of myths. 'Community mobilization and awareness' comprises subthemes such as the organization of community health events and the distribution of culturally appropriate educational materials. For 'Community acceptance and compliance', subthemes involved the role of community leaders in advocating for vaccination and the integration of vaccination programs into existing community structures. The role of healthcare workers and local leaders emerged as the most cited subtheme, reflecting the pivotal role of these individuals in promoting vaccine uptake [52,53]. The use of credible information sources was identified as the second most prominent subtheme, stressing the importance of reliable information dissemination in vaccination campaigns [43]. Another subtheme was 'Simplification of the vaccine registration process', suggesting that ease of access to vaccination services significantly impacts participation rates [43]. Other notable subthemes, such as 'quality training' and 'effective supervision and monitoring', highlight the importance of well-trained personnel and robust oversight in facilitating vaccination efforts [42]. The 'Active participation of community in vaccination programs' and 'Government support' [45,51], 'Involvement of religious leaders', and 'Improved CHW (Community Health Workers) training' [46,47,50] also emerged as subthemes

Themes for Perceived Barriers

The most prevalent themes included misinformation and lack of knowledge, fear of side effects, and doubts about vaccine efficacy. Other significant themes included pandemic fatigue, mistrust in vaccines, and accessibility issues. The themes for the barriers are presented in Table 4. Concerns about side effects and doubts about efficacy were next in frequency, indicating a strong undercurrent of vaccine skepticism. Another theme was limited staff and space/contraceptive method targets for clinics [49].

**Table 4:** Themes for barriers to interventions

Themes	Verbatims
Misinformation and lack of knowledge/Fear of side effects/Doubts about vaccine efficacy	A participant during the vaccination clinics - "I haven’t taken the vaccine because I am scared of the unknown nature of the side effects." Also another participant during the Covid vaccination - "I am unable to use the COWIN app; I will have to ask my son to help me" [43].
Pandemic fatigue/Mistrust in vaccine efficacy/Accessibility issues	A Medical officer of a low-performing PHC - "People think that there is no more COVID-19. Therefore, why to get 4–5 days fever after taking precaution dose unnecessarily. Even the focus on vaccination drive is diluted." ASHA worker vaccinated and belonging to a low performing district, - "Some even climbed up the tree to avoid vaccination. Later we convinced them. Village Administrative Officer, police, block medical officer and we all went to talk to them. We even told them they would not get their ration if they did not get the vaccine" [53].
Poor stock management/Incomplete knowledge amongst health functionaries/Fear of vaccine wastage	Field worker's observation on training gaps was that "Lack of adequate training and information at the field level about Hep B vaccine and its schedule leads to lower coverage." Health worker's concern on vaccine wastage was that "Fear of vaccine wastage due to opening a new vial for only a few beneficiaries is a significant barrier to administering the birth dose" [42].
Utilitarian Approach of Public Health Authorities/Misinformation and Fear/Coercive Vaccination Strategies	A healthcare provider - "Misinformation and rumours about vaccine safety spread faster than the facts, creating barriers to vaccination acceptance." A community Leader -: "The coercive approach in vaccination drives, without proper community engagement, often leads to resistance and noncompliance" [52].
Accessibility to services/Indirect costs/Loss of wages	A comment emerged about distance and transport to healthcare facilities about the accessibility to immunization services in terms of distance and transport to healthcare facilities. Another person - “most of the slums were not seen as legal communities (nonnotified) and therefore were not allocated government funded healthcare clinics"[45].
Financial cost/Lack of awareness	A Clinician in middle-class site settings - "People are unaware of influenza vaccination during pregnancy, but that most of them know about TT vaccination." A Woman from slum area who was a Community member - "In Government hospital, they don’t tell anything. In a private hospital, it is like we give money; therefore, they tell us" [48].
Logistics and cost concerns/Lack of knowledge about HPV	Mother aged 28 years - "When we say to them that they need to get children vaccinated, they say that they have work. In addition, even sometimes, if the parents show interest, then the government sister will not come on time promptly to the village. The parents had to wait all day and lost work for that day and lost one day wages because of the government sister’s delay." In the wake of the discussion, a summery was brought up as a cause for concern that "Primary obstacles to vaccination were related to missing work for vaccine appointments and associated costs" [51].
Rumours about vaccine safety/Inadequate knowledge about the campaign/Sudden planning and under preparedness	Senior Nursing Officer - "We were not given enough time to plan for the campaign-related activities like discussion with schools, community members; we were informed just a month before that such campaign is going to happen; so we faced some challenges during the campaign." A father who came for the discussion - "We received messages saying that the vaccine will have serious side effects and not to give it. Therefore, we were afraid to give it our children" [44].
Health system factors/Human resource factors/Community level factors	A healthcare worker - "Due to compulsory online registration for vaccination, people keep postponing for it." Another person who is a healthcare worker - "In rural areas, in the afternoon, people go to the field, which is more preferred than taking the vaccine, so we can’t cover the total population" [50].
Logistical challenges/Lack of detailed information	A participant commented about the logistic barriers due to distance that "My eldest daughter didn’t receive [vaccines] because the Anganwadi was far away, and [the closer one] didn’t provide vaccination at that time." Another participant commented that "I was angry because she said it’s for the children’s benefit, but she never clarified the benefits," which showed a lack of clear communication from healthcare providers [46].
Communication gaps/Logistical issues/Misinformation	A community Member commented that "Lack of clear and timely communication from health authorities sometimes created confusion and distrust among the community”. Another community healthcare worker stated that "There was a widespread belief that the vaccine was not necessarily due to the lack of COVID-19 cases in our area, which slowed down vaccination efforts initially" [47].

Subthemes for Perceived Barriers to Vaccination

The most prevalent subtheme was 'Technological barriers', which involves issues with digital access and understanding that affect vaccination uptake [42,50]. The influence of media and word-of-mouth emerged as a significant subtheme, indicating the strong impact of social and media narratives on public perception [44]. The 'availability of vaccines' and 'accessibility of vaccines' were frequently noted, reflecting logistical challenges in vaccine distribution and reach [43]. Subthemes of 'misinformation and rumours’ were prominent, illustrating widespread communication issues and the spread of inaccurate information [53]. Distance to healthcare facilities and a lack of awareness surfaced as a subtheme for the accessibility issue barrier among migrants. This demonstrated the reequipping and integration of such marginalized communities into the mainstream of healthcare [45]. In addition, concerns over privacy and nursing staff shortages emerged as a subtheme, all of which demonstrated the significance of institutional regulations and the limitations of available resources [49]. Another subtheme that has emerged is cost and misinformation, and their interplay can create a compound effect, exacerbating the challenges faced by individuals and communities [48]. The subthemes of work-related absence and lack of sexually transmitted infection (STI) information, excluding HIV/AIDS, revealed a misconception regarding the range of sexually transmitted infections and the advantages of immunization. The perceived cost associated with absence has been outweighed by the perceived advantage of immunization [51]. The logistical issues that surfaced as a subtheme were the lack of a mechanism for recording birth doses and the insufficiencies in training, official communications, and coordination [42]. Shortages of vaccines and supplies and inadequate infrastructure emerged as subthemes in availability issues, along with overburdened healthcare workers, community resistance, and misconceptions [50]. The absence of early notice from healthcare practitioners and the provision of inconsistent information were barriers due to a lack of commitment [47,52].

Interaction between facilitators and barrier themes

**Figure 2:**
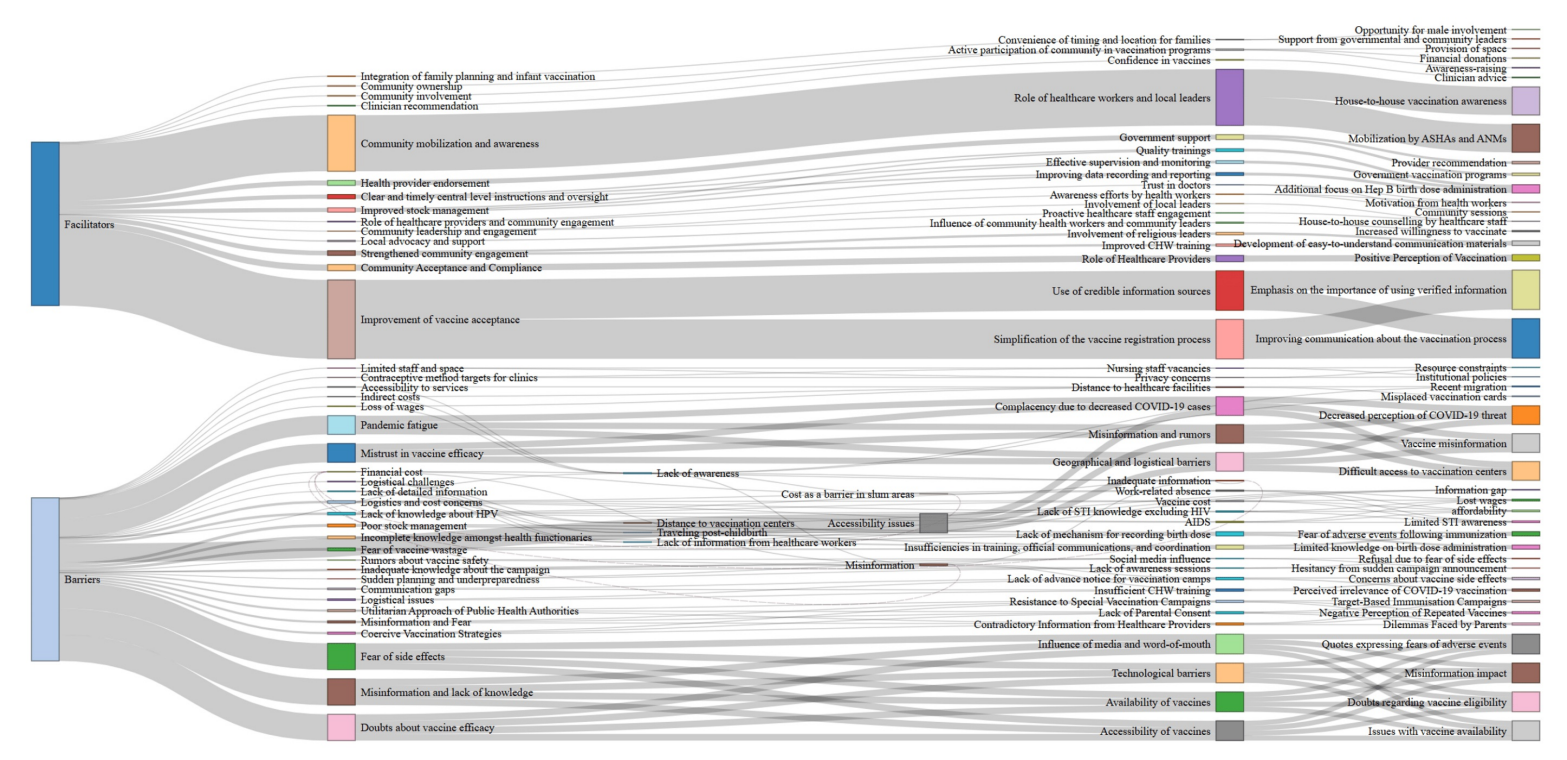
Sankey diagram portraying facilitators and barriers

A Sankey diagram is designed to illustrate the movement of data among different categories, themes, subthemes, and codes. The Sankey diagram presents a concise depiction of hierarchical connections and the allocation of factors that aid or hinder progress (Figure 2).

The following facilitators can be seen: (i) Community leaders' support: The participation of prominent community leaders had a substantial impact on fostering favourable views about immunization. Leaders' endorsements play a vital role in dispelling doubts and promoting the acceptance of vaccines in numerous communities [50]; (ii) Religious endorsements: The endorsement of vaccination by religious groups and leaders was crucial in facilitating its promotion. Religious leaders frequently address disinformation and promote vaccination by aligning it with cultural norms [47]; (iii) Cultural competence of healthcare providers: Healthcare practitioners who possessed a comprehensive understanding of and demonstrated respect for cultural practices exhibited more success in effectively conveying the advantages of vaccination, resulting in increased rates of acceptability [44,50,52].

The following barriers can be seen: (i) Misinformation and myths: The prevalence of misinformation and enduring myths regarding immunizations posed significant obstacles. These included baseless concerns regarding the safety of vaccines, misunderstandings about the components of vaccines, and suspicions of conspiracy [43,47,52]; (ii) Cultural norms and traditions: Some groups had prevalent traditional beliefs and behaviours that prevented immunization. These ideas encompassed the notion that illnesses that could be averted by vaccines were not of significant severity or that relying on natural immunity was more desirable [50,53]; (iii) Fear of side effects: Apprehensions regarding the possible negative consequences of vaccines discouraged individuals from receiving vaccinations. These anxieties are frequently intensified by anecdotal accounts and a dearth of precise information [42,47]

Sentiment Analysis in Vaccination: Beneficiaries’ and Stakeholders' Perspectives

**Figure 3:**
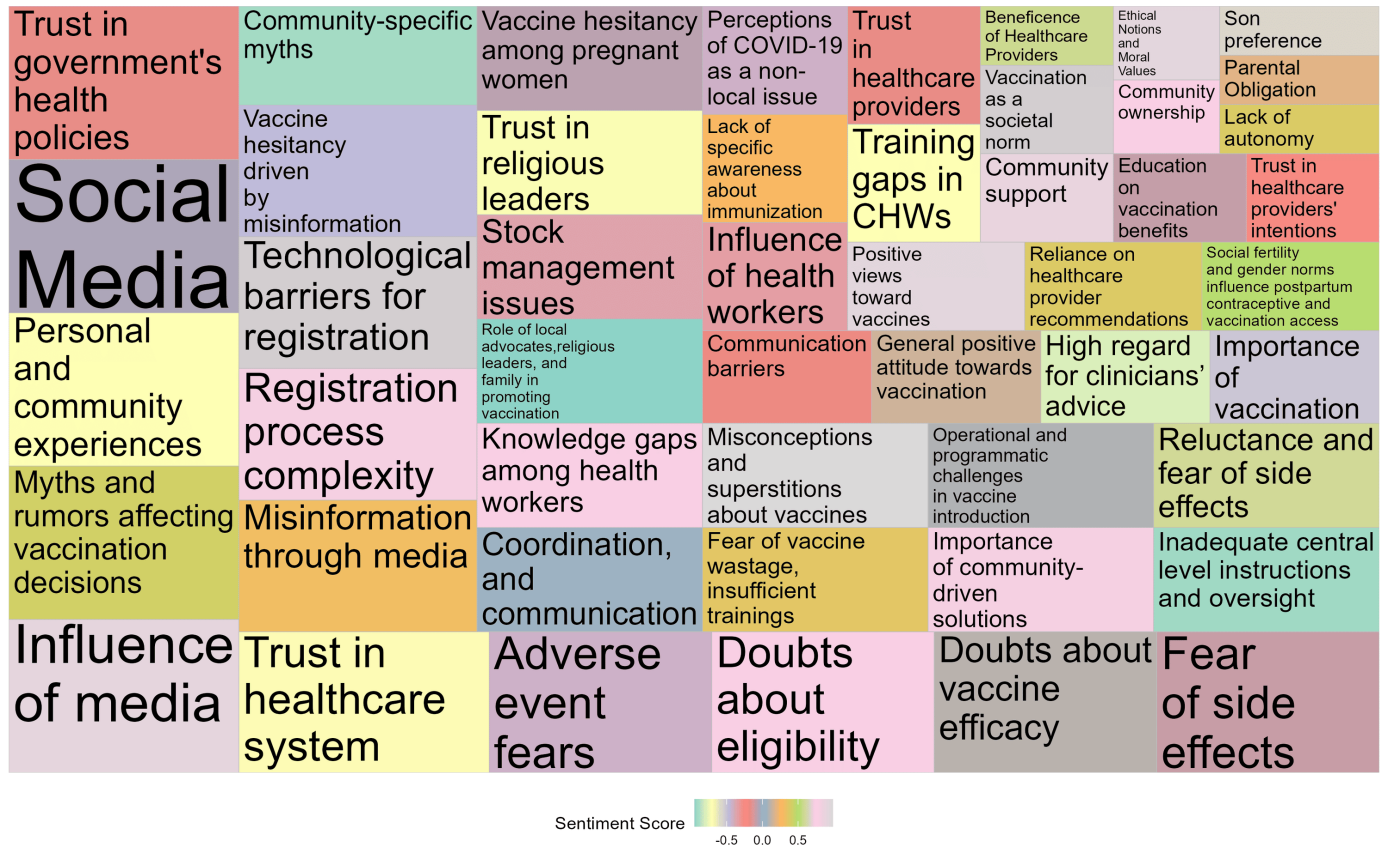
Sentiment analysis of beneficiaries and stakeholders regarding vaccination: Treemap of code frequencies and sentimental scores

The tree map, as given in Figure 3, depicts an underlying and intricate pattern of feelings regarding vaccination, where larger regions represent a higher frequency of codes. The dominant codes pertaining to 'Trust in healthcare practitioners' and 'Misinformation through media' were highly noticeable, indicating their substantial impact on vaccination opinions. The color coding represented a diverse spectrum of attitudes, spanning from profound apprehensions to resolute support for vaccination procedures. The tree map offered a graphical overview of the data, enabling the quick identification of prevailing sentiments and their respective magnitudes.

Subgroup Analysis According to Time and Place

**Figure 4:**
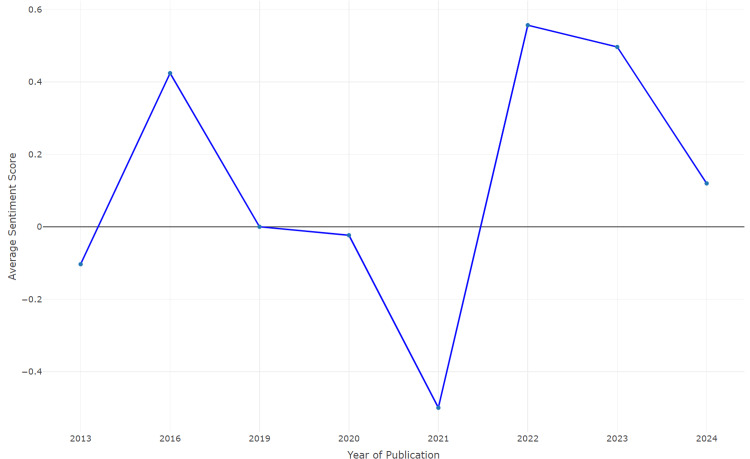
Subgroup analysis by time: Variation of sentiment scores over the years

The line plot (Figure 4) visually represents the variation in average sentiment scores over the years from 2013 to 2024. The sentiment score in 2013 started on a positive note with a score slightly above zero. This indicated generally positive sentiment towards vaccination in the early years of the dataset [51,52,54]. There was a significant peak in 2016, with the average sentiment score reaching approximately 0.4. This suggested a highly positive sentiment towards vaccination during this year, potentially due to successful vaccination campaigns or positive public health messaging [45]. The sentiment score decreased to approximately zero in 2019, indicating neutral sentiment. This could reflect a period of balanced views or mixed reactions towards vaccination during this year [44]. The sentiment score slightly decreased in 2020, indicating a small dip in positive sentiment. This might correlate with the onset of the COVID-19 pandemic and initial uncertainties surrounding the development and distribution of vaccines [48]. A noticeable trough in the sentiment score was observed in 2021, with a significant negative sentiment. This period likely reflects public concerns and controversies related to COVID-19 vaccines, including misinformation and vaccine hesitancy [43,46]. The sentiment score significantly recovered in 2023 and reached another peak similar to that in 2016, suggesting a strong positive sentiment toward vaccination [47,49,50]. This improvement could be attributed to successful vaccination rollouts and increased public confidence in vaccines. The sentiment score slightly decreased again in 2024, although it remained positive. This slight drop might indicate residual concerns or emerging issues related to vaccination [53].

**Figure 5:**
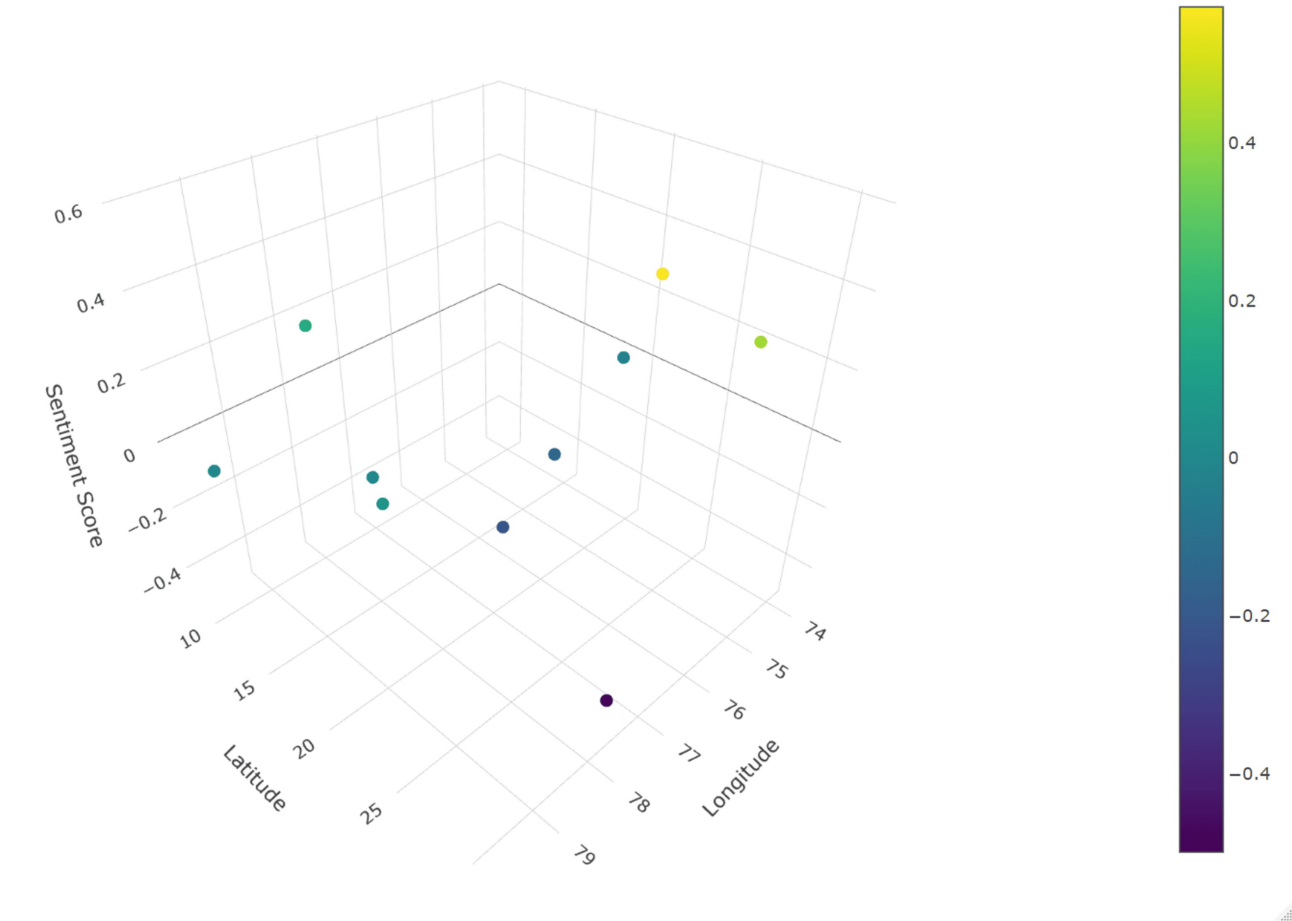
Subgroup analysis by location: three-dimensional geospatial map of sentiment score

The 3D scatter plot (Figure 5) visually represents the geospatial distribution of sentiment scores across various study areas in India. The sentiment score for Delhi [43] was slightly positive, indicating a generally positive sentiment towards vaccination in this area. The sentiment score for India is positive, suggesting an overall positive perception of vaccination across the country [53,54]. The sentiment scores for Kerala and Tamil Nadu, India, were among the highest, indicating strong positive sentiment toward vaccination. This could be reflected in effective public health campaigns and high vaccination rates in these states [52]. The sentiment score for Ludhiana was slightly negative, indicating some concerns or negative perceptions toward vaccination in this area [45]. The sentiment score for Medchal Mandal and Shamirpet Mandal, Andhra Pradesh [51] was slightly positive, reflecting a generally positive sentiment toward vaccination. The sentiment score for Mewat District, Haryana [46,47], was neutral to slightly negative, indicating mixed or uncertain perceptions of vaccination. The sentiment score for Puducherry [44] was positive, indicating favourable sentiment toward vaccination. The sentiment score for rural Maharashtra [49] is slightly negative, indicating some concerns or negative perceptions toward vaccination in rural areas. The sentiment score for the rural area of Akola district, Maharashtra [50], was neutral to slightly negative, reflecting mixed or uncertain perceptions of vaccination. The sentiment score for urban Pune [48] was positive, indicating favourable sentiment toward vaccination in urban settings.

Discussion

The variety of strategies utilized in various studies underscores the complexity of tackling immunization difficulties in India. This implies that there is no universal solution that can be applied to all situations, which means that specific solutions need to be developed for individual groups and regions. The findings highlighted the importance of cultural competence in developing and executing health interventions, as seen by the significant variation in cultural attitudes towards vaccination. Strategies that demonstrate cultural sensitivity and are customized to align with the values and beliefs of a particular community are more likely to yield positive results. The participation of diverse stakeholders, such as healthcare professionals, community leaders, and families, highlighted the importance of adopting a multistakeholder approach. This collaborative endeavour has the potential to augment the scope and efficacy of vaccination programmes, guaranteeing that interventions are both well-received and easily obtainable by the intended groups.

Facilitators of Intervention to Improve Vaccination Coverage

The quantitative synthesis identified many beneficiary-related themes, such as improvement in vaccine acceptance; community-related themes, such as community mobilization and awareness, community acceptance and compliance, community ownership/community involvement, and strengthened community engagement and community leadership and engagement. Healthcare provider-related themes include improved stock management/clear and timely central-level instructions and oversight, health provider endorsement, clinician recommendations, and the role of healthcare providers and community engagement. Other themes include local advocacy and support, and the integration of family planning and infant vaccination. The major subthemes that emerged included the use of credible information sources, simplification of the vaccine registration process, quality training/effective supervision and monitoring, active participation of community religious leaders, and improved community health worker (CHW) training.

Batteux et al. reported that improving vaccine acceptance through communicating the effectiveness and safety of the vaccine acts as a facilitator [54]. Studies have shown that reminders, in terms of print and email messages describing various aspects of vaccines, have improved vaccine acceptance among beneficiaries [55,56]. Studies have indicated that health service assistance has the most significant impact, followed by health education and discussion, follow-up and reminders, social marketing initiatives, and community mobilization [57]. By addressing the underlying factors that influence acceptance, such as trust, education, accessibility, cultural sensitivity, and misinformation, health authorities can create a more favourable environment for vaccination. This, in turn, leads to higher vaccination rates, better herd immunity, and overall improved public health outcomes [58-60].

Community engagement is a process that requires actively involving and motivating many partners to collaborate to utilize the potential of the community and improve community health. This process includes increasing awareness, mobilizing resources, fostering ownership, and encouraging involvement. The levels of community participation are organized on a spectrum that ranges from providing information and seeking input to actively involving, partnering with, and empowering the community. Community engagement is a dynamic process that involves numerous players and operates within distinct contexts to solve different concerns through several channels rather than being a single solution. Studies have shown that community-related support plays a prominent role in global immunization strategies, as it has the capacity to alleviate vaccination hesitancy and enhance vaccination confidence [57,60-65]. Moore et al. reported that a healthcare provider's suggestions play a crucial role in promoting immunization [66]. Vaccination confidence is determined by the level of trust individuals have in the safety and efficacy of vaccines, as well as their faith in healthcare experts, public health institutions, and governments responsible for establishing vaccination mandates [67]. Studies have shown that vaccination coverage is upheld through trust in healthcare providers with clear logistics [67-72], patient-provider conversations [73-76], transparent explanation of risks and benefits [75], and positively framed, action-oriented messaging that plays a proactive role in vaccine acceptance [77].

The utilization of community health workers (CHWs) in health-related programs has been successful for many years, mostly because of their perceived role as intermediaries between beneficiaries and stakeholders. They are frequently regarded as irreplaceable cadre because of their crucial role in accessing underserved communities, mitigating health inequalities, and narrowing the gap. Research indicates that adopting a positive strategy, utilizing the expertise of CHWs to create interventions, and including important individuals in the home and society, not just mothers, can help maintain improvements in health in areas with low vaccine coverage. Proactive engagement of community leaders, focused counselling, community assemblies, and home visits seem to enhance vaccine awareness and alleviate apprehensions over its safety and effectiveness [78-83]. With its increasing popularity, social media has the capacity to serve as a valuable and current source of easily accessible public health information [84]. Researchers have used targeted online sentiment analyses to assess the effectiveness of vaccine marketing activities or other prominent health programs and make necessary adjustments to efficiently distribute important information [85-88]. Research has demonstrated that the utilization of unreliable sources has diminished health literacy, particularly among younger individuals [89].

Barriers to Vaccination Coverage

The analysis identified many themes that can be categorized into system-related, informational, and logistic-related barriers. System-related barriers include the utilitarian approach of public health authorities, accessibility to services, sudden planning and underpreparedness, coercive vaccination strategies, contraceptive method targets for clinics, and human resource factors/community-level factors. Information-related barriers included misinformation and fear, a lack of awareness, a lack of knowledge about vaccines, rumours about vaccine safety, inadequate knowledge about the campaign, and a lack of detailed information and communication gaps. Logistic-related factors include limited staff and space, logistical challenges, indirect costs, and loss of wages.

The presence of concerns over potential adverse reactions and uncertainties regarding the effectiveness of vaccines indicates a lack of confidence that must be resolved by focused communication efforts by reliable healthcare authorities. The presence of accessibility challenges and inadequate stock management reveals underlying inefficiencies within the system, indicating a requirement for improved allocation of resources and management of logistics. The existence of psychological obstacles such as pandemic weariness and mistrust signifies the psychological burden of extended health crises and their influence on public health behaviours. There is a clear need for enhanced health communication techniques, which might be achieved by actively involving the community and leveraging local health staff as champions for immunization. The analysis indicates that policymakers and public health practitioners should develop comprehensive intervention strategies that address both the distribution of accurate information and the enhancement of systemic and logistical aspects of vaccination administration. There is a high demand for interventions on the basis of evidence customized to specific situations, considering the cultural and geographical variations across India.

Razai et al. reported that interactions with healthcare professionals were a major barrier to the uptake of vaccines among pregnant women [90]. The reduced vaccine uptake was attributed to pressure from practitioners, one-sided concerns, and inadequate explanations. Furthermore, healthcare professionals’ lack of clarity in providing explanations and information played a role in the decreased acceptance of the vaccine. The skepticism of individuals toward a vaccination program, whether from anti-government attitudes or concerns about the pharmaceutical sector, has also impacted their decision not to receive immunizations [73,74,77,91-95].

Esposito et al. demonstrated that significant obstacles within the healthcare system included financial constraints, challenges in vaccine storage, limited availability and delivery of vaccines, absence of an effective technique for consolidation, and missed chances for vaccination [96]. Rainey et al. [97], along with others [98,99], demonstrated a strong correlation between under-vaccination in low- and middle-income countries and health system-related barriers. These barriers include inadequate access to services, long distances to immunization centres, insufficient knowledge and attendance of health workers, and limited vaccine availability. Common logistics obstacles include the financial burden of clinical visits and vaccines, limited physical accessibility due to geographic and functional proximity to vaccination sites, restricted job flexibility for caregivers of children or older adults who are unable to take time off for vaccination, and disruptions in the supply chain, such as constraints on vaccine production, distribution, and delivery [100,101]. Kaufman et al. identified six distinct categories of obstacles that parents encounter regarding child vaccination, including issues related to accessibility, the clinic or health system, worries and beliefs, health perceptions and experiences, knowledge and information, and social or familial influence [102]. Multiple studies have demonstrated the significance of knowledge and understanding, which can empower individuals to make informed decisions regarding vaccinations. Erroneous information generates a feeling of discomfort, resulting in reluctance to access resources. The dissemination of false information through social media, as well as the lack of trustworthy internet sources, insufficient media coverage, and conflicting messages, has the potential to exacerbate this issue and negatively impact the adoption of accurate information [92,92,103-106].

Beliefs and Practices Regarding Vaccination in India

The analysis revealed various beliefs/practices that affected vaccination uptake, including social fertility and gender norms, positive health-related attitudes, trust in the healthcare system, prior experiences, misconceptions regarding vaccination, and moral/ethical notions supported by the community.

The interplay between social fertility and gender norms significantly influences individuals' choices regarding immunization. Patriarchal structures frequently influence healthcare decisions, including immunizations, in numerous Indian communities. Traditionally, men make health-related decisions, and this might influence the order in which women and children receive immunizations. Studies have shown that women's low social status manifests at every level as a barrier to accessing vaccinations; access to education, income, and autonomous decision-making about time and resource allocation are evident barriers. Women's subordinate position indirectly renders them susceptible to being held responsible and stigmatized in the event of childhood illness. While this situation has exacerbated difficulties in obtaining necessary resources, it has also heightened women's determination to employ all available methods to ensure the well-being of their children. However, in environments where gender discrimination is particularly prevalent, simply improving access and providing information may not be sufficient to reach those who are not adequately immunized [107-113].

A positive health-related attitude and trust in the healthcare system are critical factors influencing vaccination uptake. Communities that value health and well-being are more likely to accept vaccinations. Communities with greater trust in healthcare providers and institutions are more likely to participate in vaccination programs. Positive or unpleasant healthcare experiences strongly influence future healthcare behaviours, including vaccination. Negative vaccination experiences can dissuade people, whereas positive experiences can encourage them. Trust is a three-pronged model involving vaccines, institutions, and people [114]. Research has indicated that the personal attributes discussed, including both sociodemographic factors (such as race, religion, and education) and behavioural characteristics (such as knowledge, perceptions, and experience), ultimately affect the level of authority and vulnerability required for trust and building relationships [115-121].

Vaccination efforts can be either facilitated or hindered by moral and ethical convictions, which are frequently shaped by community leaders and local cultural norms. Certain groups may perceive vaccination from a moral or ethical standpoint, linking it to concepts of hygiene, integrity, or religious convictions. Parents are motivated by the perception that vaccination is a customary and obligatory responsibility they have towards their children. This issue is crucial, as it guarantees the public health department's utilitarian objective of sufficient safeguarding against diseases that can be prevented through vaccination. The ethical principles function distinctively in the process of policy-making and in the provision of services, or at the individual household level. Interventions that prioritize utility can potentially weaken parental responsibility. Put simply, state-led initiatives in immunizations are considered acceptable as long as they do not eclipse the family ideals of promoting the well-being of their children [122,123].

Strengths of the Review

The systematic review and meta-analysis included 12 studies, comprising both mixed-method and qualitative studies, which offered a wide range of data. This detailed synthesis helped us understand the elements that influence vaccination acceptance in India. Research using mixed methods and qualitative methods provides a variety of data sources and perspectives, improving the study. Multiple methods increase the reliability and completeness of the results. Employing thematic categories and subcategories to examine facilitators and barriers yielded a systematic analysis of the elements that impact the adoption of vaccination. By integrating sentiment analysis of the viewpoints of beneficiaries and stakeholders, we gained a more nuanced comprehension of the emotional and attitudinal aspects of vaccination acceptance. The cultural context of vaccination in India can be learned from beliefs, myths, and practices. Understanding these factors is essential for obtaining culturally relevant and effective responses. The subgroup analysis of location yielded region-specific insights into the factors that promote or hinder progress. The differences between urban and rural areas can be utilized to tailor solutions. Subgroup analysis enables the investigation of interactions, offering valuable insights into the influence of local healthcare infrastructure, service delivery, and healthcare provider relationships on vaccination uptake. Examining data longitudinally in subgroup analysis enables researchers to assess the effects of interventions. Researchers can evaluate the effectiveness of interventions and make data-driven decisions for future programs by comparing vaccination rates and barriers before and after their implementation.

Limitations of the Review

The search was restricted to publications published exclusively in English as a result of limited time, human, and financial resources to translate works published in other languages; this may have constrained the applicability of this review. Qualitative studies are fundamentally subjective, as they depend on the self-reported data provided by participants and the interpretations made by researchers. The presence of subjectivity in this context has the potential to generate bias, which in turn can impact the dependability of the findings. This study focused on many interventions that were diverse in terms of vaccines, providers, and methods, which may have influenced the outcomes. The restricted temporal period of the articles may have resulted in the omission of relevant discoveries from previous periods.

Recommendations

Effective vaccination interventions must involve active community engagement, which includes collaborating with community leaders, utilizing local media, and organizing community meetings to discuss and address vaccination concerns. Interventions should be culturally sensitive and tailored to the specific needs of different communities. This involves understanding local cultural practices, beliefs, and languages, and incorporating these into vaccination promotion strategies. Providing education through trusted sources and in understandable formats such as visual aids, storytelling, and community testimonials can be powerful tools in dispelling myths and reinforcing positive attitudes towards vaccination.

Addressing gender norms requires gender-sensitive approaches that empower women and involve men in conversations about vaccinations. Programs should aim to create an environment where women’s health is prioritized and where men support vaccination efforts. Building and maintaining trust in the healthcare system is fundamental. This can be achieved through consistent, quality care, transparent communication, and demonstration of the benefits of vaccination through success stories and visible health improvements in the community. Interventions should focus on addressing health decision-making norms by involving both men and women and encouraging collaborative decision-making within families.

Public health campaigns should focus on reinforcing positive health attitudes, emphasizing the benefits of vaccination in preventing diseases and improving overall community health. Building and maintaining trust through transparent communication, culturally competent care, and reliable service delivery is essential for successful vaccination interventions.

## Conclusions

The review examined both facilitators and barriers, along with beliefs and practices related to vaccine coverage efforts in India. The study identified facilitator beneficiary-related themes such as improvements in vaccine acceptance, community-related themes such as community mobilization and awareness, community acceptance and compliance, and community ownership/community involvement. Healthcare provider-related themes included improved stock management/clear and timely central-level instructions and oversight, and clinician recommendations.

The identified barriers included system-related barriers, such as the utilitarian approach of public health authorities, accessibility to services, sudden planning, underpreparedness, and coercive vaccination strategies. Information-related barriers included misinformation and fear, lack of awareness, and lack of knowledge about vaccines. Logistic-related factors included limited staff and space, logistical challenges, and costs. The beliefs and practices that affect vaccination uptake include social fertility and gender norms, positive health-related attitudes, trust in the healthcare system, prior experiences, misconceptions regarding vaccination, and moral/ethical-driven notions supported by the community.

## Appendices

Appendix A 

**Table 5:** PRISMA 2020 checklist PRISMA: Preferred Reporting Items for Systematic Reviews and Meta-Analyses

Section and Topic	Item #	Checklist item	Location where item is reported
TITLE			
Title	1	Identify the report as a systematic review.	Page 1
ABSTRACT			
Abstract	2	See the PRISMA 2020 for Abstracts checklist.	Page 1-2
INTRODUCTION			
Rationale	3	Describe the rationale for the review in the context of existing knowledge.	Page 3-5
Objectives	4	Provide an explicit statement of the objective(s) or question(s) the review addresses.	Page 5
METHODS			
Eligibility criteria	5	Specify the inclusion and exclusion criteria for the review and how studies were grouped for the syntheses.	Page 6-7
Information sources	6	Specify all databases, registers, websites, organisations, reference lists and other sources searched or consulted to identify studies. Specify the date when each source was last searched or consulted.	Page 6
Search strategy	7	Present the full search strategies for all databases, registers and websites, including any filters and limits used.	Page 6
Selection process	8	Specify the methods used to decide whether a study met the inclusion criteria of the review, including how many reviewers screened each record and each report retrieved, whether they worked independently, and if applicable, details of automation tools used in the process.	Page 6-7
Data collection process	9	Specify the methods used to collect data from reports, including how many reviewers collected data from each report, whether they worked independently, any processes for obtaining or confirming data from study investigators, and if applicable, details of automation tools used in the process.	Page 7-8
Data items	10a	List and define all outcomes for which data were sought. Specify whether all results that were compatible with each outcome domain in each study were sought (e.g. for all measures, time points, analyses), and if not, the methods used to decide which results to collect.	Page 8-9
	10b	List and define all other variables for which data were sought (e.g. participant and intervention characteristics, funding sources). Describe any assumptions made about any missing or unclear information.	Page 8-9
Study risk of bias assessment	11	Specify the methods used to assess risk of bias in the included studies, including details of the tool(s) used, how many reviewers assessed each study and whether they worked independently, and if applicable, details of automation tools used in the process.	Page 7
Effect measures	12	Specify for each outcome the effect measure(s) (e.g. risk ratio, mean difference) used in the synthesis or presentation of results.	NA
Synthesis methods	13a	Describe the processes used to decide which studies were eligible for each synthesis (e.g. tabulating the study intervention characteristics and comparing against the planned groups for each synthesis (item #5)).	Page 8-9
	13b	Describe any methods required to prepare the data for presentation or synthesis, such as handling of missing summary statistics, or data conversions.	Page 8-9
	13c	Describe any methods used to tabulate or visually display results of individual studies and syntheses.	Page 8-9
	13d	Describe any methods used to synthesize results and provide a rationale for the choice(s). If meta-analysis was performed, describe the model(s), method(s) to identify the presence and extent of statistical heterogeneity, and software package(s) used.	Page 8-9
	13e	Describe any methods used to explore possible causes of heterogeneity among study results (e.g. subgroup analysis, meta-regression).	Page 8-9
	13f	Describe any sensitivity analyses conducted to assess robustness of the synthesized results.	NA
Reporting bias assessment	14	Describe any methods used to assess risk of bias due to missing results in a synthesis (arising from reporting biases).	NA
Certainty assessment	15	Describe any methods used to assess certainty (or confidence) in the body of evidence for an outcome.	Page 7, additional

			file 3 and 4
RESULTS			
Study selection	16a	Describe the results of the search and selection process, from the number of records identified in the search to the number of studies included in the review, ideally using a flow diagram.	Page 9-10
	16b	Cite studies that might appear to meet the inclusion criteria, but which were excluded, and explain why they were excluded.	Page 9-10, figure 1
Study characteristics	17	Cite each included study and present its characteristics.	Page 9-10, table 1 and 2
Risk of bias in studies	18	Present assessments of risk of bias for each included study.	Page 7, additional file 3 and 4
Results of individual studies	19	For all outcomes, present, for each study: (a) summary statistics for each group (where appropriate) and (b) an effect estimate and its precision (e.g. confidence/credible interval), ideally using structured tables or plots.	Table 1 and 2
Results of syntheses	20a	For each synthesis, briefly summarise the characteristics and risk of bias among contributing studies.	Additional file 3 and 4
	20b	Present results of all statistical syntheses conducted. If meta-analysis was done, present for each the summary estimate and its precision (e.g. confidence/credible interval) and measures of statistical heterogeneity. If comparing groups, describe the direction of the effect.	Page 10-16 Table 3 and 4
	20c	Present results of all investigations of possible causes of heterogeneity among study results.	Page 10-16
	20d	Present results of all sensitivity analyses conducted to assess the robustness of the synthesized results.	Figure 4 and 5
Reporting biases	21	Present assessments of risk of bias due to missing results (arising from reporting biases) for each synthesis assessed.	Page 9-16
Certainty of evidence	22	Present assessments of certainty (or confidence) in the body of evidence for each outcome assessed.	Page 9-16
DISCUSSION			
Discussion	23a	Provide a general interpretation of the results in the context of other evidence.	Page 16-24
	23b	Discuss any limitations of the evidence included in the review.	Page 24
	23c	Discuss any limitations of the review processes used.	Page 24
	23d	Discuss implications of the results for practice, policy, and future research.	Page 25
OTHER INFORMATION			
Registration and protocol	24a	Provide registration information for the review, including register name and registration number, or state that the review was not registered.	Page 2 and 5
	24b	Indicate where the review protocol can be accessed, or state that a protocol was not prepared.	Page 5
	24c	Describe and explain any amendments to information provided at registration or in the protocol.	NA
Support	25	Describe sources of financial or non-financial support for the review, and the role of the funders or sponsors in the review.	Page 26
Competing	26	Declare any competing interests of review authors.	Page 27

interests				
Availability of data, code and other materials	27	Report which of the following are publicly available and where they can be found: template data collection forms; data extracted from included studies; data used for all analyses; analytic code; any other materials used in the review.		Additional file 2, 3, 4

Appendix B 

**Table 6:** Search strategy Search Dates: January 2013 to December 2024

Code	Search Strategy
#1	“vaccination” [Title/Abstract] OR “national health policies”[Title/Abstract] OR “national health program”[Title/Abstract] OR “primary health care”[Title/Abstract] OR “intervention”[Title/Abstract] OR “health programs”[Title/Abstract] OR “health insurance”[Title/Abstract] OR “health care system”[Title/Abstract] OR “community health”[Title/Abstract] OR “health services”[Title/Abstract] OR “public health”[Title/Abstract] AND “accessibility of health services”[Title/Abstract] OR “accessibility, program”[Title/Abstract] OR “facilitators barriers”[Title/Abstract] OR “influencing factors”[Title/Abstract] OR “predictors”[Title/Abstract] OR “health care utilization”[Title/Abstract] OR “utilization indicators”[Title/Abstract] OR “health evaluation”[Title/Abstract] OR “risk benefit assessment”[Title/Abstract] AND “india”[Title/Abstract] AND "qualitative"[Title/Abstract]
#2	“vaccination” [Title/Abstract] OR “health policy” [Title/Abstract] OR “health program” [Title/Abstract] OR “primary health” [Title/Abstract] OR “intervention” [Title/Abstract] OR “health insurance” [Title/Abstract] OR “public health care system” [Title/Abstract] OR “community health” [Title/Abstract] OR “health service utilization” [Title/Abstract] OR “accessibility” [Title/Abstract] OR “facilitators barriers” [Title/Abstract] OR “indicators” [Title/Abstract] OR “health evaluation” [Title/Abstract] OR “risk benefit” [Title/Abstract] AND “india” [Title/Abstract] AND "qualitative" [Title/Abstract]
#3	#1 OR #2
#4	(((((((((((((((((((vaccination[MeSH Terms]) OR (national health program[MeSH Terms])) OR (primary health care[MeSH Terms])) OR (intervention)) OR (health programs)) OR (health insurance)) OR (health care system)) OR (community health)) OR (health services)) OR (public health)) AND (accessibility of health services[MeSH Terms])) OR (accessibility, program[MeSH Terms])) OR (facilitators barriers)) OR (influencing factors)) OR (predictors)) OR (health care utilization)) OR (utilization indicators)) OR (health evaluation)) OR (risk benefit assessment[MeSH Terms])) AND (india)) AND (qualitative))
#5	#1 OR #4
#6	#2 OR #4

Appendix C 

**Table 7:** CASP checklist CASP:  Critical Appraisal Skills Programme; HPV: human papillomavirus

Sl. No.	Study Title	C1	C2	C3	C4	C5	C6	C7	C8	C9	C10
1	Understanding feasibility and acceptability of implementation of linking delivery of family planning and infant vaccination care in rural Maharashtra India: a qualitative study	Y	Y	Y	Y	Y	Y	Y	Y	Y	Y
2	Evaluation of a community-based intervention to mprove routine childhood vaccination uptake among migrants in urban slums of Ludhiana, India	Y	Y	Y	Y	Y	Y	Y	Y	Y	Y
3	Antenatal influenza vaccination in urban Pune India: clinician and community stakeholders’ awareness priorities and practices	Y	Y	Y	Y	Y	Y	Y	Y	Y	Y
4	Facilitators and barriers to the uptake of COVID-19 vaccine precaution dose among adult population: qualitative analysis across six different states of India	Y	Y	Y	Y	Y	Y	Y	Y	Y	Y
5	Acceptability of HPV Vaccine Implementation Among Parents in India	Y	Y	Y	Y	Y	Y	Y	Y	Y	Y
6	An Assessment of Hepatitis B Vaccine Introduction in India: Lessons for Roll out and Scale up of New Vaccines in Immunization Programs	Y	Y	Y	Y	Y	Y	Y	Y	Y	Y
7	Factors related to vaccine hesitancy during the implementation of Measles-Rubella campaign 2017 in rural Puducherry-A mixed-method study	Y	Y	Y	Y	Y	Y	Y	Y	Y	Y
8	Barriers and Facilitators of COVID-19 Vaccination Outreach Program in Rural India: A Qualitative Study	Y	Y	Y	Y	Y	Y	Y	Y	Y	Y
9	Caregiver perceptions of the broader societal benefits of vaccination: A path toward sustainable vaccine advocacy in India	Y	Y	Y	Y	Y	Y	Y	Y	Y	Y
10	Leading from the frontlines: community-oriented approaches for strengthening vaccine delivery and acceptance	Y	Y	Y	Y	Y	Y	Y	Y	Y	Y
11	The interactions of ethical notions and moral values of immediate stakeholders of immunisation services in two Indian states: a qualitative study	Y	Y	Y	Y	Y	Y	Y	Y	Y	Y
12	COVID Appropriate Behaviour compliance and Vaccine Hesitancy: Findings from a COVID-19 Health Education Campaign in a Government Tertiary Care Hospital in Delhi, India	Y	Y	Y	Y	Y	Y	Y	Y	Y	Y

Appendix D 

**Table 8:** SRQR checklist SRQR: Standards for Reporting Qualitative Research; HPV: human papillomavirus

Study Title	S 1	S2	S3	S4	S5	S6	S7	S8	S9	S 1 0	S 1 1	S 1 2	S 1 3	S 1 4	S 1 5	S 1 6	S 1 7	S 1 8	S 1 9	S 2 0	S 2 1
Understanding feasibility and acceptability of implementation of linking delivery of family planning and infant vaccination care in rural Maharashtra India: a qualitative study	Y	Y	Y	Y	Y	Y	Y	Y	Y	Y	Y	Y	Y	Y	Y	Y	Y	Y	Y	Y	Y
Evaluation of a community- based intervention to improve routine childhood vaccination uptake among migrants in urban slums of Ludhiana, India	Y	Y	Y	Y	Y	Y	Y	Y	Y	Y	Y	Y	Y	Y	Y	Y	Y	Y	Y	N	Y
Antenatal influenza vaccination in urban Pune India: clinician and community stakeholders’ awareness priorities and practices	Y	Y	Y	Y	Y	Y	Y	Y	Y	Y	Y	Y	Y	Y	Y	Y	Y	Y	Y	Y	Y
Facilitators and barriers to the uptake of COVID-19 vaccine precaution dose among adult population: qualitative analysis across six different states of India	Y	Y	Y	Y	Y	N	Y	Y	Y	Y	Y	Y	Y	Y	Y	Y	Y	Y	Y	Y	Y
Acceptability of HPV Vaccine Implementation Among Parents in India	Y	Y	Y	Y	Y	N	Y	Y	Y	Y	Y	Y	Y	Y	Y	Y	Y	Y	Y	N	Y
An Assessment of Hepatitis B Vaccine Introduction in India: Lessons for Roll out and Scale up of New Vaccines in Immunization Programs	Y	Y	Y	Y	Y	Y	Y	Y	Y	Y	Y	Y	Y	Y	Y	Y	Y	Y	Y	Y	Y
Factors related to vaccine hesitancy during the implementation of Measles- Rubella campaign 2017 in rural Puducherry-A mixed-method study	Y	Y	Y	Y	Y	Y	Y	Y	Y	Y	Y	Y	Y	Y	Y	Y	Y	Y	Y	Y	N
Barriers and Facilitators of COVID-19 Vaccination Outreach Program in Rural India: A Qualitative Study	Y	Y	Y	Y	Y	Y	Y	Y	Y	Y	Y	Y	Y	Y	Y	Y	Y	Y	Y	Y	N
Caregiver perceptions of the broader societal benefits of vaccination: A path toward sustainable vaccine advocacy in India	Y	Y	Y	Y	Y	Y	Y	Y	Y	Y	Y	Y	Y	Y	Y	Y	Y	Y	Y	Y	Y
Leading from the frontlines: community-oriented approaches for strengthening	Y	Y	Y	Y	Y	Y	Y	Y	Y	Y	Y	Y	Y	Y	Y	Y	Y	Y	Y	Y	Y
